# Electrocardiographic findings in patients with sickle cell disease: A protocol for systematic review and meta‐analysis

**DOI:** 10.1002/hsr2.2212

**Published:** 2024-06-23

**Authors:** Alireza Sadeghi, Ehsan Taherifard, Hamed Movahed, Alireza Ahmadkhani, Niloofar Dehdari Ebrahimi, Erfan Taherifard

**Affiliations:** ^1^ Student Research Committee Shiraz University of Medical Sciences Shiraz Iran; ^2^ Hematology Research Center Shiraz University of Medical Sciences Shiraz Iran; ^3^ Department of Pathology Shiraz University of Medical Sciences Shiraz Iran; ^4^ Transplant Research Center Shiraz University of Medical Sciences Shiraz Iran

**Keywords:** electrocardiography, meta‐analysis, sickle cell disease, systematic review

## Abstract

**Background:**

Despite advancements in the management of patients with sickle cell disease (SCD), the involvement of the cardiovascular system in these patients remains a significant concern. Cardiovascular manifestations of SCD are well‐documented, with electrocardiography (ECG) serving as a valuable diagnostic tool. Studies have reported a high rate of critical ECG findings in patients with SCD that warrants consideration when managing these patients, indicating the need for proactive cardiac screening and management strategies in this patient population. This study aims to systematically review the literature to identify sociodemographic, clinical, and paraclinical factors associated with ECG abnormalities in patients with SCD.

**Methods:**

A comprehensive search strategy will be employed across multiple online databases, including PubMed, Embase, Scopus, Web of Science, and Google Scholar, for published and gray literature. Eligible studies will include original articles reporting associations between sociodemographic, clinical, and paraclinical variables and a spectrum of ECG findings in patients with SCD. Independent reviewers will conduct the screening, quality assessment, and data extraction. Quantitative analyses will be performed under a random‐effect model using Comprehensive Meta‐Analysis software, with subgroup analyses based on SCD status, sickle hemoglobinopathy form, and age group.

## 1 INTRODUCTION

Sickle cell disease (SCD) encompasses a group of hereditary red blood cell disorders that impose a substantial burden on affected individuals and healthcare systems due to the chronic nature of the condition and its associated complications.^1^, ^2^ Despite advancements in improving the survival rates of individuals with SCD over the years, studies have consistently shown that their average life expectancy remains significantly lower than that of the general population.^3^, ^4^, ^5^, ^6^ This disparity in life expectancy can be attributed to various factors, with cardiac complications and sudden cardiac death emerging as prominent contributors.^7^, ^8^ The cardiovascular manifestations of SCD have been extensively studied, spanning from the underlying pathophysiology to the clinical outcomes associated with cardiac involvement.^9^, ^10^, ^11^, ^12^ The involvement of the cardiovascular system in these patients is multifactorial, primarily attributed to chronic ischemic, inflammatory, and oxidative stressors, endothelial dysfunction, and structural remodeling in cardiac tissues. These pathophysiological processes contribute to the development of various cardiac abnormalities, including myocardial ischemia and infarction, diastolic and systolic dysfunction, pulmonary hypertension, and valvular abnormalities.^13^, ^14^, ^15^

Moreover, these cardiovascular system involvements have been shown to manifest in the electrocardiograms (ECGs) of patients with SCD, a noninvasive and cost‐effective tool that provides valuable insights into the cardiac function and electrical activity of the heart. In a previous systematic review and meta‐analysis conducted by our team, we comprehensively assessed the spectrum and pattern of ECG abnormalities observed in this patient population.^16^ By analyzing data from 59 studies, we found that three‐quarters of patients with SCD exhibit various ECG abnormalities with significantly higher rates compared to healthy individuals. Notably, these ECG abnormalities include several critical findings, warranting consideration when managing these patients, such as ventricular hypertrophy, prolonged corrected QT (QTc) interval, and ST depression.

This significantly higher prevalence of ECG abnormalities in individuals with SCD highlights the critical need for proactive cardiac screening and management strategies in this patient population. This imperative is further underscored by the heightened risk of cardiovascular mortality, all‐cause mortality, and drug adverse events in patients with these abnormalities.^7^, ^12^, ^17^, ^18^, ^19^, ^20^, ^21^, ^22^, ^23^, ^24^ Early detection and management of these ECG abnormalities are, therefore, essential for optimizing patient outcomes and enhancing overall survival rates. Furthermore, understanding the sociodemographic, clinical, and paraclinical factors associated with these ECG abnormalities could provide valuable guidance for risk stratification and targeted interventions for patients with SCD. In this study, our objective is to systematically review the literature to identify and analyze these associations, thereby informing clinical practice and guiding future research efforts aimed at improving the management and outcomes of individuals with SCD.

## 2 MATERIALS AND METHODS

The Preferred Reporting Items for Systematic Reviews and Meta‐Analysis (PRISMA) 2020 statement will be used to transparently and precisely report this study.^25^ To maintain transparency and preclude bias, we have also registered the study protocol in the International Prospective Register of Systematic Reviews (PROSPERO) with ID of CRD42023446917.

### 2.1 Systematic search

In this investigation, we will employ several online databases to ensure a thorough review of existing literature in the field. These online databases will be MEDLINE (via PubMed), Embase, Scopus, Web of Science, and Google Scholar. We will combine two sets of terms related to SCD and ECG to have a sensitive yet specific systematic search. “Sickle cell,” “Sickle cell anemia,” “Sickle cell trait,” “Sickle cell syndrome,” “Sickle cell disease,” “hemoglobin S disease,” “HBS disease,” and “Sickling disorder” were terms in the first group which was related to SCD and “ECG,” “EKG,” “Electrocardiogram,” “Electrocardiographic,” and “Electrocardiography” were the terms of the other group. Our search for ECG‐related terms will not be limited to the title, abstract, and keywords of the articles to ensure no articles are missed. The detailed search strategy we designed for each database is available in the Supporting Information Material.

We conducted the comprehensive search strategy initially on October 7, 2023, and will repeat it just before finalizing the systematic review and meta‐analysis to include the latest relevant research findings. Alongside our thorough search in the online databases, we will manually and meticulously examine the reference lists from the identified records. This manual review aims to identify any additional sources or studies worthy of consideration, thus reducing potential publication bias in our systematic review.

### 2.2 Eligibility criteria

This study will include original articles providing detailed information on the association between sociodemographic, paraclinical, and clinical variables and a spectrum of ECG findings in patients with SCD. These ECG findings may encompass abnormalities such as arrhythmias, structural abnormalities, conduction irregularities, as well as abnormalities in intervals, segments, and waves. We will not impose restrictions on the types of effect sizes utilized to signify the presence or absence of an association between various variables and ECG abnormalities. Various effect sizes, spanning from correlation and regression coefficients to mean differences and proportional measures such as relative risk or hazard ratios, are all considered eligible.

Besides, there will be no restrictions on the study designs eligible for inclusion in this meta‐analysis, thereby encompassing both observational and clinical trial studies, provided they contain relevant data. The eligible study population will be individuals across all ranges of age, encompassing both children and adults, who have been diagnosed with any form of sickle hemoglobinopathies. These various forms of sickle hemoglobinopathies may encompass the homozygous state of HbS (sickle cell anemia), the heterozygous state of HbS and HbA (sickle cell trait), or double heterozygosity of HbS and another hemoglobinopathy, such as β thalassemia or HbC. Additionally, no constraints will be imposed regarding the publication date, the language of the articles, the site of patient recruitment, whether in outpatient clinics, hospital settings, or other healthcare facilities, or the type of articles such as comments, correspondences, and conference papers.

The exclusion criteria will cover studies where ECG reports are from nonstandardized ECG acquisitions, encompassing instances where ECG data collection deviates from established protocols or employs unconventional methodologies. This includes the use of single or segmental leads limited to either precordial or limb leads. Additionally, studies reliant on unconventional ECG settings, such as those conducted under experimental conditions like exercise or hypoxic environments, will also be considered ineligible for inclusion. Furthermore, studies employing nontraditional methods for ECG analysis, such as computer‐generated interpretations without human validation, will also be excluded. Lastly, we will exclude studies narrowly focused on specific subgroups of patients with SCD, such as those with pulmonary hypertension, comorbid conditions like diabetes mellitus or chronic kidney disease, or pregnant individuals with SCD.

### 2.3 Selection process and data extraction

Following the systematic search across online databases, the yielded citation records will be imported into Endnote Library (Clarivate Analytics) version X9. Upon import, any duplicate records will be automatically identified using the library's duplicate finding feature and subsequently removed. The screening process, comprising two rounds, will then commence. These screening rounds will be independently conducted by two researchers from our team. During the first round, the title, abstract, and keywords of each record will be thoroughly assessed against the predetermined eligibility criteria. In the second round, the full‐text of each record will undergo careful evaluation. Any disagreements regarding the inclusion or exclusion of specific records will be resolved through discussion with the corresponding author.

Following the identification of the eligible articles, the data extraction process will be initiated and conducted independently by two researchers. Excel spreadsheets will serve as the medium for data extraction. The extracted details will encompass various parameters, including the first author's name, publication date, the country where the study was conducted, patient recruitment setting, number of SCD patients, number of participants in the control group, characteristics of patient and control groups, percentage of males in each group, the age distribution of participants in each group, SCD status (steady‐state or sickle cell crisis), type of sickle hemoglobinopathy, specified associations between sociodemographic, paraclinical, and clinical variables and a spectrum of ECG findings assessed in the study, along with their respective effect sizes, if applicable.

### 2.4 Risk of bias assessment

For the observational studies included in our analysis, we will evaluate their quality using the Newcastle−Ottawa scale checklist corresponding to their specific design.^26^ In the case that an included article is a clinical trial, we will employ Cochrane tools to assess the risk of bias. This assessment will depend on the design of the clinical trial, with the revised Cochrane risk of bias tool for randomized trials (RoB2) for randomized trials and the risk of bias in non‐randomized studies of interventions (ROBINS‐I) for nonrandomized studies.^27^, ^28^ Similar to the prior process, this step will also be independently conducted by two researchers.

### 2.5 Statistical analysis

Comprehensive Meta‐Analysis software (Biostat Inc.) version 3 will be used for quantitative analyses. All the analyses will be conducted under the DerSimonian−Laird random‐effect model.^29^ Various outcome measures of associations reported across studies will be entered into the software for effect size calculation. These effect sizes may include mean differences, odds ratios, hazard ratios, or correlation coefficients, depending on the nature of the association. The pooled effect sizes and their corresponding 95% confidence intervals will be estimated to assess the overall magnitude and direction of the associations between the variables and ECG abnormalities. The results of the quantitative analyses will be visually represented using forest plots. Statistical heterogeneity across the included studies in each quantitative analysis will be evaluated using the *I* ^2^ statistic and Cochran's *Q* test. Subgroup analyses will be conducted based on SCD status, the form of sickle hemoglobinopathy, and the age group of the participants for each association. Sensitivity analyses will be performed using the leave‐one‐out method. Publication bias will be evaluated using funnel plots and Egger's test. A *p*‐value less than 0.05 was considered statistically significant.

## AUTHOR CONTRIBUTIONS

The study was conceptualized by Ehsan Taherifard, Niloofar Dehdari Ebrahimi, and Erfan Taherifard. Alireza Sadeghi, Hamed Movahed, and Alireza Ahmadkhani contributed to the methodology. Drafting of the protocol was undertaken by Ehsan Taherifard, Hamed Movahed, and Alireza Ahmadkhani. The draft was revised and edited by Alireza Ahmadkhani, Niloofar Dehdari Ebrahimi, and Erfan Taherifard. All authors have reviewed and approved the final manuscript.

## CONFLICT OF INTEREST STATEMENT

The authors declare no conflict of interest.

## TRANSPARENCY STATEMENT

The lead author, Erfan Taherifard, affirms that this manuscript is an honest, accurate, and transparent account of the study being reported, that no important aspects of the study have been omitted, and that any discrepancies from the study as planned (and if relevant, registered) have been explained.

## Supporting information

Supporting information.

## Data Availability

Data sharing is not applicable to this article as no data sets were generated or analyzed during the current study.
