# Supplementary material for: Electrocardiographic findings in patients with sickle cell disease: A protocol for systematic review and meta‐analysis
Source: Health Sci Rep. 2024 Jun 23;7(6):e2212. doi: 10.1002/hsr2.2212 (PMC11194293; doi:10.1002/hsr2.2212)
Supplement: Supplementary file 1 — Supporting information. [file HSR2-7-e2212-s001.pdf]

1 KEYWORDS

ECG	Sickle cell
EKG	sickle cell disease
Electrocardiogram	Sickle cell syndrome
Electrocardiographic	Sickle cell anemia
Electrocardiography	Sickle cell trait
	hemoglobin S disease
	HBS disease
	Sickling disorder

2 PUBMED

((((((((((Sickle cell[Title/Abstract])) OR (Sickle cell disease[Title/Abstract])) OR (Sickle cell syndrome[Title/Abstract])) OR (Sickle cell anemia[Title/Abstract])) OR (Sickle cell trait[Title/Abstract])) OR (hemoglobin S disease[Title/Abstract])) OR (HBS disease[Title/Abstract])) OR (Sickling disorder[Title/Abstract])) OR (anemia, sickle cell[MeSH Terms])) AND ((((((ECG) OR (EKG)) OR (Electrocardiogram)) OR (Electrocardiographic)) OR (Electrocardiography)) OR (electrocardiography[MeSH Terms]))

3 WEB OF SCIENCE

(Sickle cell (Topic) or Sickle cell disease (Topic) or Sickle cell syndrome (Topic) or Sickle cell anemia (Topic) or Sickle cell trait (Topic) or hemoglobin S disease (Topic) or HBS disease (Topic) or Sickling disorder (Topic) AND ECG (All Fields) or EKG (All Fields) or Electrocardiogram (All Fields) or Electrocardiographic (All Fields) or Electrocardiography (All Fields))

4 SCOPUS

((TITLE-ABS-KEY (sickle AND cell)) OR (TITLE-ABS-KEY (sickle AND cell AND disease)) OR (TITLE-ABS-KEY (sickle AND cell AND syndrome)) OR (TITLE-ABS-KEY (sickle AND cell AND anemia)) OR (TITLE-ABS-KEY (hemoglobin AND s AND disease)) OR (TITLE-ABS-KEY (hbs AND disease)) OR (TITLE-ABS-KEY (sickling AND disorder)) OR (TITLE-ABS-

KEY (sickle AND cell AND trait))) AND ((ALL (ecg)) OR (ALL (ekg)) OR (ALL (electrocardiogram)) OR (ALL (electrocardiographic)) OR (ALL (electrocardiography)))

5 EMBASE

('sickle cell':ti,ab,kw OR 'sickle cell disease':ti,ab,kw OR 'sickle cell syndrome':ti,ab,kw OR 'sickle cell anemia':ti,ab,kw OR 'sickle cell trait':ti,ab,kw OR 'hemoglobin s disease':ti,ab,kw OR 'hbs disease':ti,ab,kw OR 'sickling disorder':ti,ab,kw) AND (ecg OR ekg OR electrocardiogram OR electrocardiographic OR electrocardiography)
